# Thyroid hormone – triiodothyronine – has contrary effect on proliferation of human proximal tubules cell line (HK2) and renal cancer cell lines (Caki-2, Caki-1) – role of E2F4, E2F5 and p107, p130

**DOI:** 10.1186/1756-6614-1-5

**Published:** 2008-10-13

**Authors:** Piotr Poplawski, Alicja Nauman

**Affiliations:** 1 Department of Biochemistry and Molecular Biology, The Medical Centre of Postgraduate Education, Warsaw, Poland

## Abstract

**Background:**

Triiodothyronine regulates proliferation acting as stimulator or inhibitor. E2F4 and E2F5 in complexes with pocket proteins p107 or p130 stop cells in G1, repressing transcription of genes important for cell cycle progression. p107 and p130 inhibits activity of cyclin/cdk2 complexes. Expression of all those proteins could be regulated by triiodothyronine. In clear cell renal cell carcinoma many disturbances in T3 signaling pathway was described, in that type of cancer also expression of some key G1 to S phase progression regulators was shown.

**Methods:**

We investigated role of T3 and its receptors in regulation of proliferation of HK2, Caki-2, Caki-1 cell lines (cell counting, cytometric analysis of DNA content) and expression of thyroid hormone receptors, E2F4, E2F5, p107 and p130 (western blot and semi-quantitative real time PCR). Statistical analysis was performed using one-way ANOVA.

**Results and Conclusion:**

We show that T3 inhibits proliferation of HK2, and stimulates it in Caki lines. Those differences are result of disturbed expression of TR causing improper regulation of E2F4, E2F5, p107 and p130 in cancer cells.

## Background

Thyroid hormone – triiodothyronine – regulates cells differentiation, proliferation and programmed death. Triiodothyronine acts both as an activator and inhibitor of cells proliferation [1]. Thyroid hormones act mainly by their nuclear receptors, which are ligand dependent transcription factors. Depending on type of TRE (thyroid hormone response elements) they could activate or inactivate transcription of TH regulated genes [2]. There are evidence that T3/TR negatively regulates E2F1 transcription [3]. However, it has been also reported TRβ receptors inhibits cells proliferation independently on triiodothyronine [4,5].

E2F4 and E2F5 belongs to E2F transcription factors family. Those two E2Fs do not have nuclear localization signal and their binding to promoters depends on interactions with p107 and p130 [6]. p107 and p130 belong to the family of retinoblastoma suppressor proteins [7]. E2F4 or E2F5 in complexes with p107 or p130 when connected, take part in formation of CERC (cyclin E repressor complex). In G1 phase CERC binds to *CCNE1 *promoter repressing transcription, and inhibiting proliferation. Phosphorylation of p107 and p130 by kinases results in CERC dissociation and activation *CCNE1 *transcription and cell cycle progression [8]. Other cell cycle regulatory genes are regulated in the same way. p107 and p130 in addition to creating CERC, could inhibit proliferation also by inhibiting the activity of cyclin E/cdk2 and cyclinA/cdk2 complexes [9].

At least 3% of human cancers are kidney cancers. Clear cell renal cell carcinoma (ccRCC) is the most common subtype of renal cancer, representing 75–82% of primary malignancies of the kidney [10,11]. T3 signaling pathway is disturbed in that type of cancer – patients with ccRCC suffer from LTS (low triiodothyronine syndrome) [12], expression of type I deiodinase mRNA and its activity are lowered in ccRCC [13], expression of TRα1 and TRβ1 is disturbed [14,15], lowered biding of TR to DNA [16], lowered biding of T3 to TR [15,17] and mutations of TR [16]. Those disturbances frequently result in improper G1/S phase progression [18]. Elevated levels of E2F1 and cyclin E1 and its LMW were described in ccRCC [16,17,19].

Basing on those facts we decided to investigate influence of triiodothyronine on cell proliferation and expression of TRs, E2F4, E2F5, p107 and p130. That type of triiodothyronine action was never examined in kidney and ccRCC cells, and the results could shed the light, how the disturbances in T3 signaling pathway affect cell proliferation in ccRCC.

## Methods

### Cell culture, maintenance, synchronization, and flow cytometric assay

HK2 – human proximal tubules cell line, Caki-2 – human ccRCC cell line, Caki-1 human ccRCC skin metastasis. Cells were cultured in 75 cm2, 25 cm2 cell culture flasks and 6 or 12 well plates (Corning-Star) in (HK2) KSFM medium (Gibco) or (Caki-2, CAki-1) Mc'Coy 5A Medium (Gibco) supplemented with 10% Charcoal-Dextran treated FBS (Hyclone), antibiotics (Penicillin-Streptomycin solution Sigma) and with or without 100 nM triiodothyronine. Cells were synchronized by 24 h serum depletion. For growth curves and flow cytometry cells were harvested by trypsynization and counted in Malassez chamber every two hours. For expression assays, western blot and flow cytometry cells were harvested by trypsynization at three points of cell cycle G1, G1/S and S (at 4th, 8th and 12th hour after synchronization for HK2; at 18th, 22nd and 26th for Caki-2; at 12th, 16th and 20th for Caki-1). For flow cytometric analysis synchronized cells were harvested and fixed in 70% ethanol. DNA content was established by method described by Fan [20] on FACSCalibur (Becton Dickinson) briefly, cells were washed with PBS, treated with RNase (Sigma; 500 units/ml) at 37°C for 15 min, and stained with propidium iodide (Sigma; 50 μg/ml).

### Isolation of cell lysates

Cells were lysed for 30 minutes in 100 μl ice cold buffer containing 50 mM Tris (pH7,4), 150 mM NaCl, 0,5 % NP40, 50 mM NaF, 1 mM Na3VO4 1 mM DTT, 1 mM PMSF, protease inhibitor cocktail (SIGMA) and 10 μg/ml trypsin inhibitor. Then lysates were centrifuged at 18000 g for 5 min. Supernatants divided into 30 μl aliquots and stored at -70°C. The amount of protein in supernatant was established by Bradford method.

### Isolation of nuclear protein

The buffers used for isolation were as described by Kane [21]. All buffers were supplemented with protease inhibitor cocktail (SIGMA) and PMSF (0.5 mM). Cell were suspended in 1 ml of ice-cold STM buffer (20 mM Tris-HCl, 0.25 M sucrose, 1.1 mM MgCl2; pH 7.85) and centrifuged at 1000 g for 10 min at 4°C. The resulting pellet was washed twice in 1 ml of STM buffer with 0.5% Triton X-100, followed by centrifugation under the same conditions. The final pellet was resuspended in 0.1 ml KSTM + 20% glycerol buffer (20 mM Tris, 0.25 M sucrose, 1.1 mM MgCl2, 0.4 mM KCl, 20% glycerol; pH 7.85), sonicated and incubated on ice for 30 minutes with vortexing every 5 min to extract the soluble nuclear proteins. After solubilization, the suspension was centrifuged at 12000 g for 15 min at 4°C. The amount of protein in supernatant was established by Bradford method. The protein extracts were divided into 30 μl aliquots and stored at -70°C.

### Western blot analysis

40 μg of cell lysate or nuclear protein extract was resolved on 10% polyacrylamide gel (SDS-PAGE). After electrophoresis proteins were transferred onto nitrocellulose membrane. Destained membranes were blocked overnight at 4°C in 5% non-fat milk in TBS-T buffer (20 mM Tris-HCl, 137 mM NaCl, 1 M HCl, 0.1% Tween-20; pH 7.6), washed twice in TBS-T for 10 min at RT, then incubated for 2 h with mouse monoclonal anti-TR antibody (1 μg/ml TBS-T, Santa Cruz Biotechnology), mouse monoclonal anti-TRβ antibody (2 μg/ml TBS-T, NeoMarkers), mouse monoclonal anti-p107 antibody (1 μg/ml TBS-T, Santa Cruz Biotechnology) or mouse anti-actin antibody (1:10000) for 1 h at RT, washed 3 times for 10 min in TBS-T, incubated for 1 h at RT with horseradish peroxidase-conjugated goat anti-mouse secondary antibody (1:2000 in TBS-T, Dako), and washed again as described above. The proteins were detected by an enhanced-chemiluminescence detection system (Supersignal West Pico chemiluminescent substrate, PIERCE) according to standard procedures. The amount of the specific protein was estimated from densitometry after normalization against β-actin band.

### RNA isolation

Cells were suspended in 1,5 ml TRIZOL reagent (Invitrogen), incubated for 5 minutes at RT. Then 300 μl chloroform was added and mixed gently, incubated for 10 minutes and centrifuged for 15 minutes at 12000 g (4°C). Upper aqueous phase was transferred to a new tube, mixed with 0.5 ml isopropyl alcohol and incubated for 10 minutes at RT, centrifuged for 15 minutes at 12000 g (4°C). RNA pellet was washed with 75% ethanol, dried and resuspended in 50 μl DEPC-treated water.

### Real-time PCR

The isolated RNA served as a substrate in reverse transcription reaction and the obtained cDNA samples were used in semi-quantitative real-time PCR analyzes. Reverse transcription was performed with RevertAidTM H Minus First Strand cDNA Synthesis Kit (Fermentas) using oligo-dT primer, according to manufacturer's protocol.

Real-time PCR reactions were performed with QuantiFast SYBR Green PCR Kit (Qiagen) according to manufacturer's protocol. Primers are shown at table 1. Real-time PCR conditions were as follows: initial denaturation: 95°C, 10 min., followed by 40 cycles: 95°C for 20 s, 59°C for 30 s and 72°C for 60s; then melting curve analysis. Expression levels were normalized to values obtained for HPRT.

**Table 1:** Primers for SQ-RT-PCR

gene	Primers sequences	Product size
*THRA*	GCCTTTAACCTGGATGACACG GTGTTTGCGGTGGTTGACG	153 pz

*THRB*	CCTGGGACAAACCGAAGCA GATGAGATGTGGCGACGACT	140 pz

*E2F4*	GAGTGGTCCCATTGAGGTTC GGCAGAGGTGGAGGTGTAG	129 pz

*E2F5*	TCAGGCACCTTCTGGTACAC GGGCTTAGATGAACTCGACTC	145 pz

*RBL1*	GAGGTGGTGATCCGCTCAGA TAGAGGAGACATTGGCATCAG	240 pz

*RBL2*	CAACAATGGGCAAACGGTAAC GCCACTTGACCAGGGACTTG	207 pz

*HPRT*	AGGACTGAACGTCTTGCTCG GTTGGATTATACTGCCTGACC	350 pz

### Statistical analysis

Data are presented as mean ± SEM. Statistical analysis was performed using one-way ANOVA, p < 0.05 was considered statistically significant.

## Results and Discussion

### Proliferation and cell cycle

**Figure 1:**
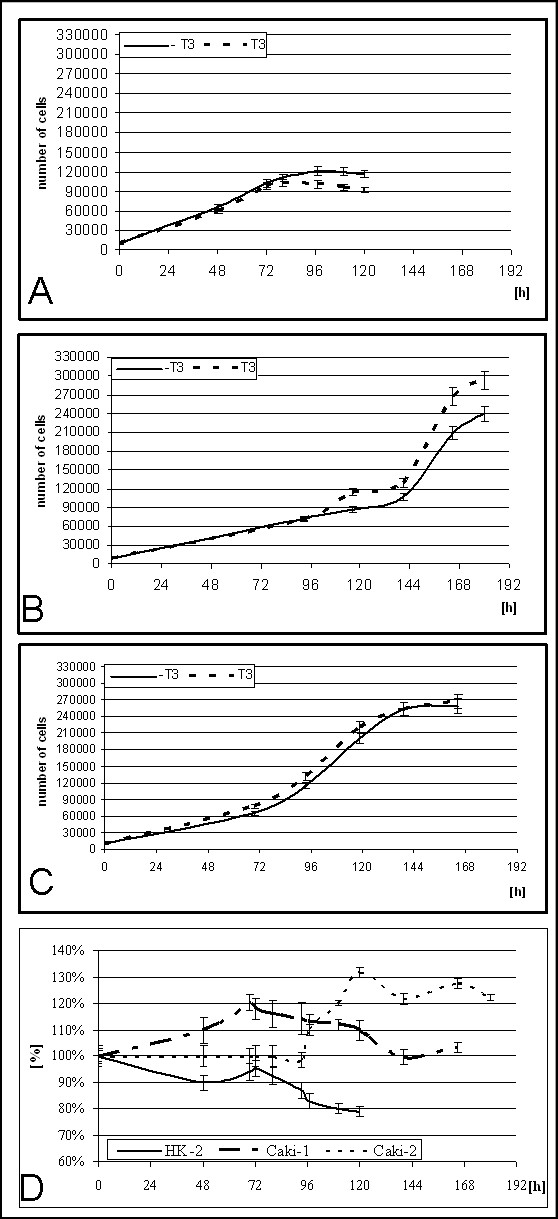
**Proliferation of cell lines**. A) growth curve for HK2 cells cultured without or with 100 nM triiodothyronine B) growth curve for Caki-2 cells cultured without or with 100 nM triiodothyronine C) growth curve for Caki-1 cells cultured without or with 100 nM triiodothyronine D) differences in proliferation caused by 100 nM triiodothyronine.

HK2 cells divided slower than cells of cancerous cell lines (Caki-2, Caki-1) (doubling time respectively 85 h, 35 h and 30 h). Triiodothyronine inhibits proliferation of HK2 cells and stimulates it in Caki-2 and Caki-1 (Fig. 1A, 1B, 1C). T3 causes 10–20% drop of cell number in HK2, and 10–20% rise of cell number was observed in Caki-2 and 20–30% at Caki-1 (Figure 1D). After synchronization from 85% (HK2) to 95%(Caki-2) cells was in G1 phase of cell cycle. HK2 cells started entering S phase about 4 hours after the end of synchronization. Maximal number of cells (20%) in S phase was observed about 12 h after end of synchronization. In the presence of T3 less HK2 cells enters S phase (5–10%). Caki-2 cells enters S phase 14 hours after synchronization, maximal number of cells in S phase (35%) was observed 28 hours after synchronization. In the presence of triiodothyronine maximal number of cells was observed from 24 to 28 hour after synchronization, more cells was entering S phase (5%). In Caki-1 cells S phase starts between 8th and 12th hour after synchronization, maximal number of cells entering S phase (40%) was observed 20 hours after synchronization. In the presence of T3 maximal number of cells in S phase rises to 50%.

It is known that proximal tubules proliferate slower than ccRCC derived cells [22], therefore HK2, Caki-2 and Caki-1 proved to be a proper cellular model for investigation of differences in cell cycle between normal and cancerous cells. Effects of triiodothyronine differs – there are evidence for proproliferative effects of that hormone [23-25] and antiproliferative action [26-28] in our experiments triiodothyronine inhibits proliferation in HK2 cells and stimulates it in cancerous cells. Results obtained from flow cytometric analysis of DNA content suggest that observed differences in cell cycle progression could be effect of inappropriate G1 to S phase progression.

### Expression of thyroid hormone nuclear receptors

**Figure 2:**
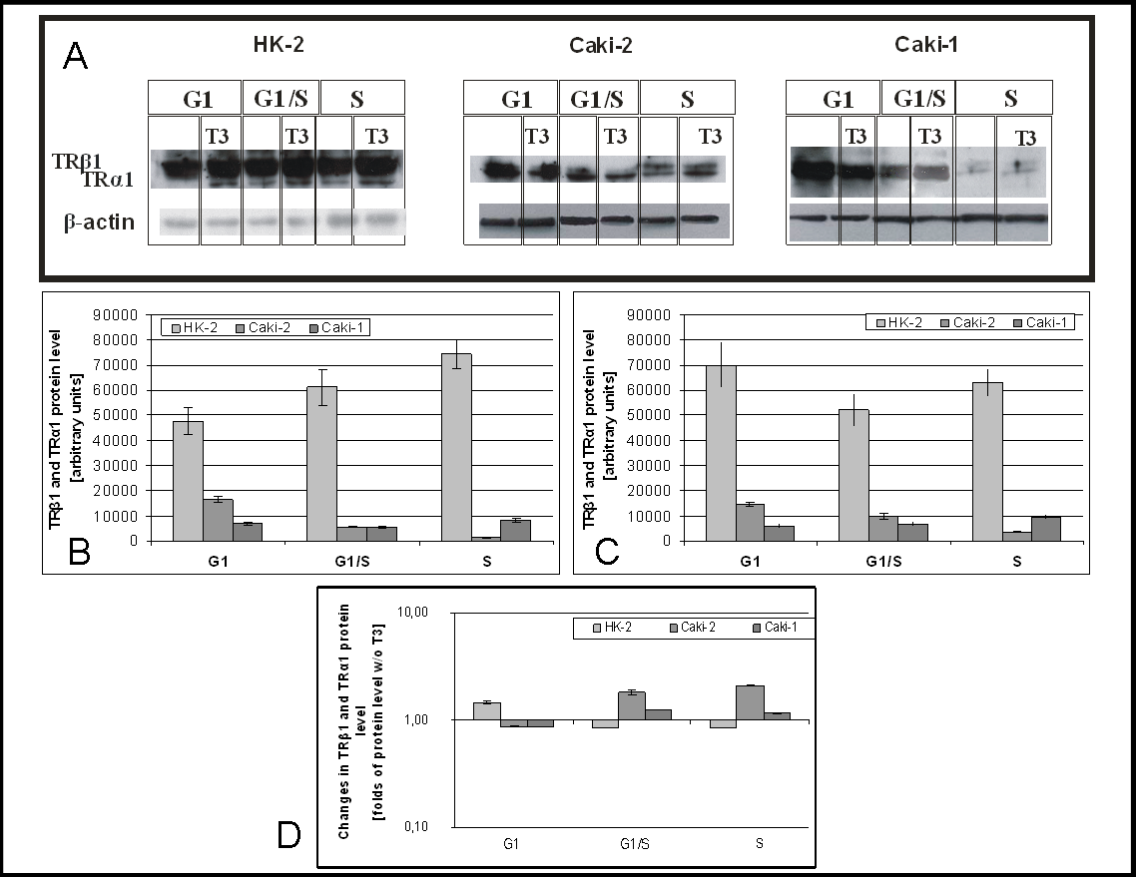
**Level of main isoforms of thyroid hormone nuclear receptors (TRβ1 and TRα1)**. A) Representative immunoblots B) mean levels of TR in cells cultured without triiodothyronine C) mean levels of TR in cells cultured with 100 nM triiodothyronine D) Changes of TR protein level caused by 100 nM triiodothyronine.

Densitometric analysis of immunoblots revealed that level of TR protein in HK2 lysates from cells cultured without rises during cell cycle progression (G1:47632 ± 5230, G1/S:61092 ± 7234, S:74248 ± 5698) (Figure 2B). In cancer cell lines Caki-2 and Caki-1 level of that proteins was lower than in HK2 cells. In Caki-2 differently than in HK2, TR protein level drops during progression of cell cycle (G1:16380 ± 1224, G1/S:5480 ± 326, S:1600 ± 172).

TR proteins level was, also in the presence of triiodothyronine, higher in HK2 than in cancerous cell lines. In HK2 TR proteins level drops at G1/S and raises at S (G1:70036 ± 8921; G1/S:52124 ± 6213; S:63044 ± 5123), In Caki-2 decreases during cell cycle (G1:14409 ± 965; G1/S:10000 ± 1234; S:3382 ± 298), and raises with cell cycle progression (G1:5957 ± 435; G1/S:6627 ± 578; S:9548 ± 789) in Caki-1 (Figure 2C).

Triiodothyronine causes raise of TR level at G1 in HK2 and decrease at G1/S and S. In Caki-2 influence of triiodothyronine was different – it caused drop at G1 and raise at G1/S and S. In Caki-1 effects of triiodothyronine on the level of TR protein was similar to that observed in Caki-2 cells (Figure 2D).

High level of TRs observed in HK2 cells confirms earlier results obtained on tissue samples of ccRCC [14]. Low level of TR in cancer cells suggests that effects of apo-receptors will weaker in those cell lines than in proximal tubules. As expected changes in level of TR proteins were observed in HK2 cells – according to the literature level of TRs rises during cell cycle progression [29]. Contrary, in Caki-2 cells expression profile was improper – protein level drops during progression.

Effects of T3 on TR level differs between HK2 and Caki-2 and Caki-1, suggesting improper action of TRs in those cell lines. Disturbances of TR could be result of different expression, but also improper action weak DNA [16] and T3 binding [15,17] or possible mutations [16].

**Figure 3:**
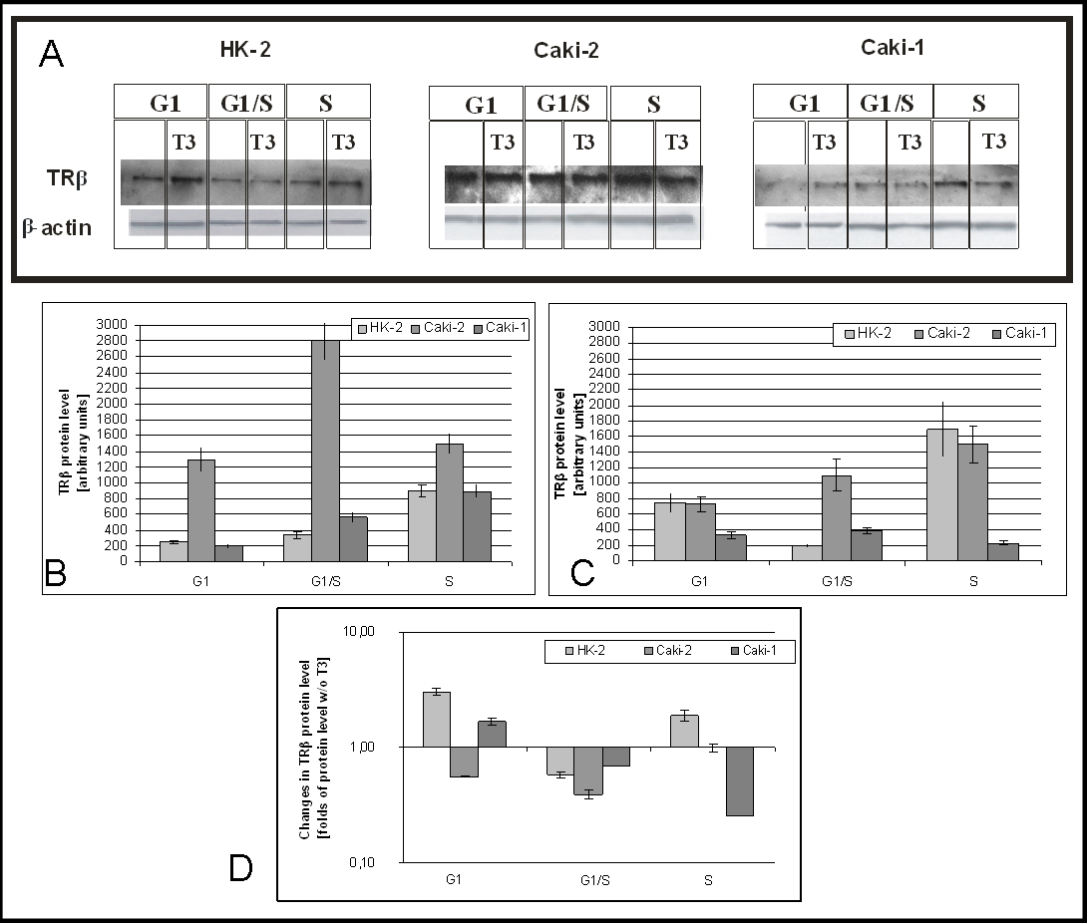
**Level of thyroid hormone nuclear receptor TRβ**. A) Representative immunoblots B) mean levels of TRβ in cells cultured without triiodothyronine C) B) mean levels of TRβ in cells cultured with 100 nM triiodothyronine D) Changes of TRβ protein level caused by 100 nM triiodothyronine.

Densitometric analysis of immunoblots show that during cell cycle progression (G1:248 ± 21; G1/S:334 ± 42; S:899 ± 78) TRβ protein level raises in nuclear extracts HK2 from cells cultured without triiodothyronine. In Caki-2 TRβ level was higher than in HK2, and was raising at G1/S (G1:1302 ± 154, G1/S:2800 ± 237) and decreasing at S. In Caki-1 raise of TR proteins level during cell cycle progression was also observed (G1:198 ± 13, G1/S:566 ± 56, S:888 ± 78) (Figure 3B).

In the presence of triiodothyronine TRβ proteins level drops at G1/S and rises at S (G1: 752 ± 120; G1/S:194 ± 14; S: 1700 ± 345) In Caki-2 TRβ level was similar to HK2 (730 ± 98, p > 0,05), and rises during cell cycle progression (G1/S:1100 ± 201, S:1500 ± 245). In Caki-1 at G1 and S TRβ level was lower than in HK2, and rises At G1/S and diminishes at S (Figure 3C).

In HK2 cell line triiodothyronine causes rise of TRβ protein level at G1 and S, and drop at G1/S. In Caki-2 at G1, effects of triiodothyronine was opposite to that observed in HK2. In Caki-1 effect of triiodothyronine was weaker than in HK2. In Caki-1 at S triiodothyronine acts as negative regulator of TRβ protein level (Figure 3D).

As opposed to the cell lysates level of TRβ in nuclear extracts was higher in Caki-2 than in HK2. Similarly Puzianowska-Kuźnicka at al [14] show raised level of TRβ1 in 30% of investigated ccRCC tissue samples. High level of TRβ could be result of more efficient transport of TRs to nucleus in Caki-2 cells. In the presence of T3 there were no differences between Caki-2 and HK2 in the level of TRβ, which suggests that transport of TRs in Caki-2 is independent of T3, its known that in normal cells transport of TRs is hormone dependent [30]. Raising during cell cycle level of TRβ suggest that effects of those receptors will be higher in later phases of cell cycle. In cancerous cell lines effects of T3 on TRβ are different than in HK2 cells. It suggest that in cancer cells expression or protein degradation is disturbed.

**Figure 4:**
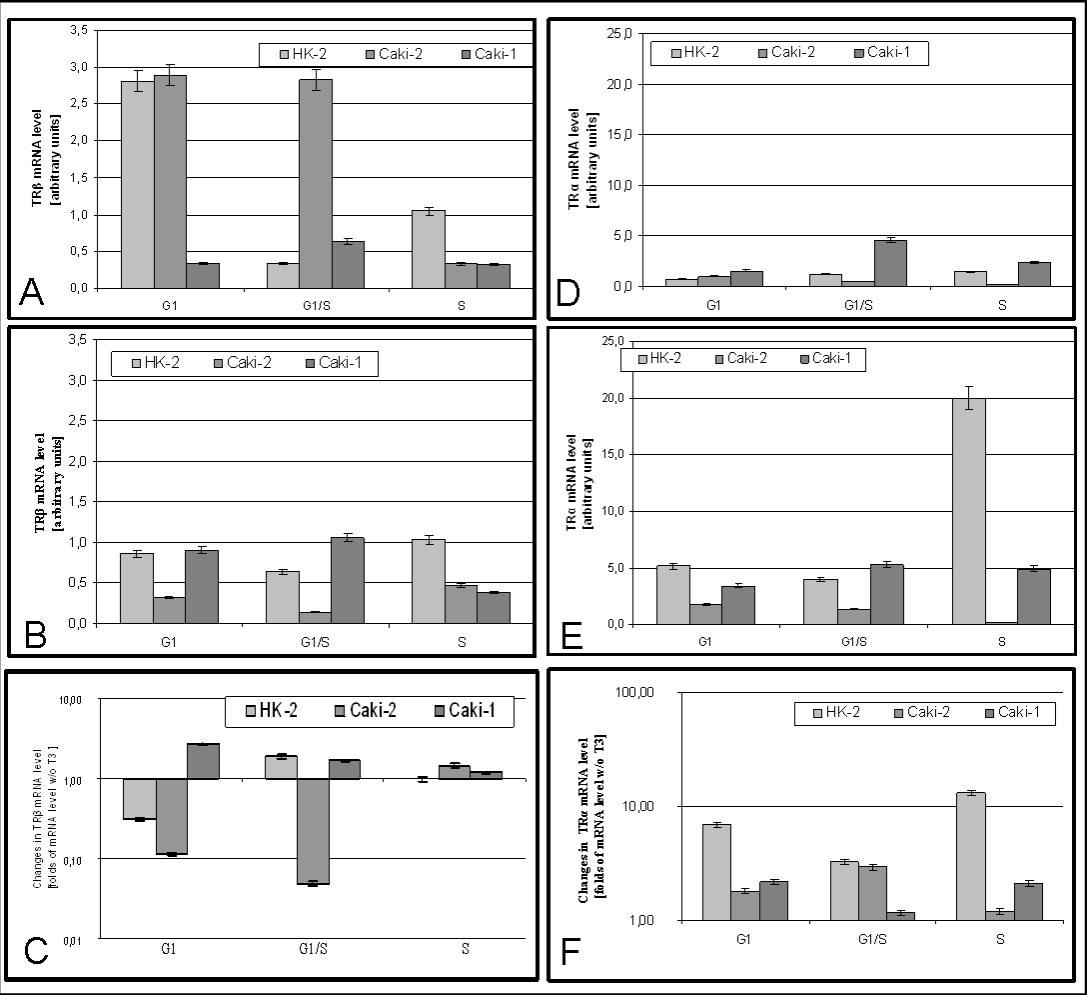
**Expression of TRβ and TRα mRNA**. A) Expression of TRβ in cells cultured without triiodothyronine B) Expression of TRβ in cells cultured with 100 nM triiodothyronine C) Changes in TRβ mRNA expression caused by 100 nM triiodothyronine D) Expression of TRα in cells cultured without triiodothyronine E) Expression of TRα in cells cultured with 100 nM triiodothyronine F) Changes in TRα mRNA expression caused by 100 nM triiodothyronine.

In HK2 cells cultured without triiodothyronine TRβ mRNA level drops at G1/S and rises at S (G1:2,9 ± 0,24; G1/S 0,3 ± 0,02; S:1,1 ± 0,05) (Figure 4A). In Caki-2 at G1 mRNA level was similar to observed in HK2 (2,8 ± 0,14), remained unchanged at G1/S (2,8 ± 0,16, p > 0,05) and decrease eight fold at S (0,33 ± 0,02). In Caki-1 level of TRβ mRNA At G1 was lower than in (0,3 ± 0,0,2 p < 0,05). In that line level of TRβ mRNA doubles at G1/S and drops at S (0,32 ± 0,03 p > 0,05).

In the presence of T3 level of TRβ mRNA in HK2 drops at G1/S and rises at S (Figure 4B). In Caki-2 level of TRβ mRNA was lower at G1 than in HK2 (G1:0,3 ± 0,02), declined at G1/S (0,1 ± 0,01) and raised at S (0,5 ± 0,02). In Caki-1 level of TRβ mRNA was similar to observed in HK2 (HK2:0,86 ± 0,04; Caki-1 0,90 ± 0,05 p > 0,05), but level of that mRNA raises at G1/S to drop at S.

In HK2 cells triiodothyronine causes negative regulation of TRβ expression at G1, positive at G1/S and has no effect at S. (Figure 4C). In Caki-2 triiodothyronine regulates expression negatively at G1 and G1/S and positively At S. In Caki-1 triiodothyronine causes rise of mRNA level, with diminishing effect over the cell cycle.

Triiodothyronine regulation of TRβ expression is different in cancerous cell lines than in HK2 line, supporting the hypothesis that TRs is disturbed in ccRCC.

In HK2 cells cultured without triiodothyronine level of TRα mRNA rises during cell cycle progression (G1:0,7 ± 0,04; G1/S:1,2 ± 0,06; S:1,5 ± 0,08) (Figure 4D). In Caki-2, differently than in HK2, level of TRα mRNA drops during cell cycle progression (G1:1,0 ± 0,05; G1/S:0,5 ± 0,02; S:0,2 ± 0,01). In Caki-1 the level of TRα mRNA was generally higher than in the other cell lines, with significant rise at G1/S and drop at S (G1:1,6 ± 0,08; G1/S:4,6 ± 0,23; S:2,3 ± 0,12).

In the presence of T3 level of TRα mRNA In HK2 drops at G1/S and rises at S (G1:5,1 ± 0,26; G1/S:3,9 ± 0,20; S:20,0 ± 1,00). In Caki-2 mRNA of this receptor was higher than in HK2 and diminishes during cell cycle progression(G1:1,8 ± 0,09; G1/S:1,3 ± 0,07; S:0,2 ± 0,01). In Caki-1 level of TRα mRNA rises at G1/S and drops at S (Figure 4E).

In all cell lines triiodothyronine causes rise of TRα mRNA level (Figure 4F).

In cells cultured without triiodothyronine expression profile of TRα is different than in Caki-2 and Caki-1 lines, suggesting that THRA expression is also disturbed in ccRCC. In the presence of T3 in HK2 and Caki-2 expression pattern of TRα was opposite, once again suggesting disturbances of TR regulation and expression in ccRCC.

### Expression E2F4 and E2F5

**Figure 5:**
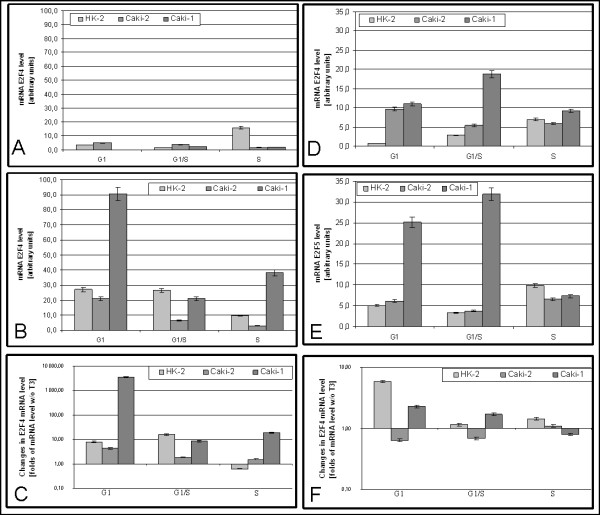
**Expression of E2F4 and E2F5 mRNA**. A) Expression of E2F4 in cells cultured without triiodothyronine B) Expression of E2F4 in cells cultured with 100 nM triiodothyronine C) Changes in E2F4 mRNA expression caused by 100 nM triiodothyronine D) Expression of E2F5 in cells cultured without triiodothyronine E) Expression of E2F5 in cells cultured with 100 nM triiodothyronine F) Changes in E2F5 mRNA expression caused by 100 nM triiodothyronine.

In HK2 cells cultured without triiodothyronine, level of E2F4 mRNA in G1/S was lower than In G1 and raised 10 folds in S (15,81 ± 0,80)(Figure 5A). In Caki-2 cells level of E2F4 mRNA was decreasing during cell cycle progression (G1: 4,96 ± 0,25; G1/S3,64 ± 0,18; S: 1,87 ± 0,09) and was statistically higher than in HK2 at G1 and G1/S and lower at S. Level of E2F4 mRNA in Caki-1 cells was increasing during cell cycle progression.

In the presence of triiodothyronine level of E2F4 mRNA in HK2 cells remains unchanged at G1 and G1/S (27,1 ± 1,36; 26,5 ± 1,32 p > 0.05) and decreases at S (9,7 ± 0,48). (Figure 5B). In cancerous line Caki-2 E2F4 mRNA level was decreasing during cell cycle progression (G1: 21,1 ± 1,01 G1/S:6,7 ± 0,34 S:2,9 ± 0,14). Caki-1 at G1 (90,5 ± 4,60) E2F4 mRNA level was higher than in HK2 and Caki-2, drops at G1/S (21,0 ± 1,05) and raises at S (38,1 ± 1,91).

In HK2 cells triiodothyronine causes raise of E2F4 level at G1 and G1/S and drop at S. In Caki-2 T3 stimulates expression of E2F4 mRNA, this effect is weakening with cell cycle progression. In Caki-1 triiodothyronine effects at G1 and S were stronger than in other cell lines.(Figure 5C).

Lower proliferation of HK2 cells could be attributed to the higher level of E2F4. Lower level of E2F4 in Caki-2 and Caki-1 lines is similar to that observed in breast [31] and thyroid cancer [32]. In Caki-2 level of E2F4 mRNA decreases during cell cycle progression it may result in faster proliferation – E2F4 is known as an inhibitor of proliferation. Lower level of E2F4 in the presence of T3 and its drop during the cell cycle explain faster proliferation of that cell line. Higher level in Caki-1 is hard to explain, but it is known that in gastric cancers mutations of E2F4 were observed [33]. For proper interpretation of those results assessment of p107 and p130 levels is also important. Effects of T3 on triiodothyronine expression were similar in all cell lines, however slightly weaker in Caki-1.

In HK2 cells cultured without triiodothyronine E2F5 mRNA level raises during cell cycle progression (G1:0,87 ± 0,04; G1/S:2,81 ± 0,014; S:6,93 ± 0,35). Caki-2 cells at G1(9,68 ± 0,48) and S (5,43 ± 0,21) points has higher level of E2F5 mRNA than in HK2. At those cells level of that mRNA drops almost two-fold at G1/S. In Caki-1 E2F5 mRNA level was higher than in other cell lines (G1:11,05 ± 0,55; G1/S:18,83 ± 0,94; S:9,25 ± 0,46). (Figure 5D).

In the presence of triiodothyronine in HK2 cells level of E2F5 mRNA falls two-times at G1/S and rises at S (G1:5,1 ± 0,25; G1/S:3,2 ± 0,16; S: 6,6 ± 0,33) (Figure 5E). Similar changes were observed in Caki-2 cells, in Caki-1 cells, at G1 E2F5 mRNA level was higher than in other cell lines, with rise during G1/S and drop at S.

Triiodothyronine causes stimulation of E2F5 expression in HK2 and this effect was strongest at G1. In Caki-2 at G1 and G1/S triiodothyronine represses E2F5 mRNA expression, stimulation observed at S was weaker than in HK2. Effects of triiodothyronine in Caki-1 were similar to those observed in HK2 (Figure 5F).

According to the literature [34] higher level of E2F5 mRNA observed in cancer cell lines, should result in proliferation inhibition, but such effect was never observed. In breast cancer amplification of E2F5 gene was observed [35]. Negative regulation of E2F5 expression by triiodothyronine in Caki-2 could be a reason of faster proliferation observed in that cell line.

### Expression of p107 and p130

**Figure 6:**
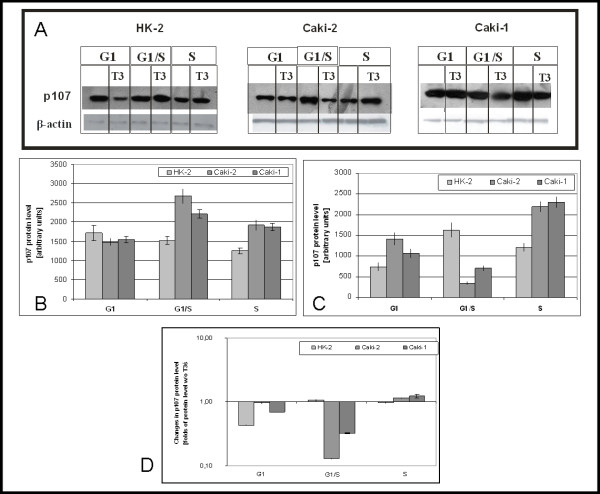
**Level of p107 protein**. A) Representative immunoblots B) mean levels of p107 in cells cultured without triiodothyronine C) mean levels of p107 in cells cultured with 100 nM triiodothyronine D) Changes of p107 protein level caused by 100 nM triiodothyronine.

Densitometric analysis of immunoblots revealed that in nuclear extracts from HK2 cells cultured without T3 level of p107 was decreasing during cell cycle progression (G1:1716 ± 200; G1/S:1525 ± 100; S:1254 ± 79) (Figure 6B). In Caki-2 level of p107 protein at G1 (1478 ± 100) was lower than in HK2, and differently than in HK2 rises at G1/S(2679 ± 187) and drops at S (1929 ± 138). In Caki-1 p107 protein level at G1 (1545 ± 150) was similar to Caki-1, at G1/S also rises (2208 ± 135) but raise was weaker, at S(1871 ± 220 p107 protein level was similar to that observed in Caki-2).

In the presence of triiodothyronine p107 protein level doubles at G1/S and drops at S (G1:742 ± 100; G1/S:1626 ± 170; S:1207 ± 97)(Figure 6C). In Caki-2 at G1 (1415 ± 150) p107 protein level was two fold higher than in HK2, dropped at G1/S(344 ± 28) and raised at S (2195 ± 120). In Caki-1 like in Caki-2, higher than in HK2 level of p107 protein was observed, changes in this protein level was similar to Caki-2.

In HK2 cells triiodothyronine causes drop of p107 level at G1 and has almost no effect at other parts of cell cycle(Figure 6D). In Caki-2 T3 has no effects at G1, but at G1/S causes nine fold drop of p107 level. In Caki-1 at G1 (similar to HK2) triiodothyronine causes drop of p107 protein level and at G1/S (similar to Caki-2) causes fall of p107 protein level.

In Caki-2 and Caki-1 cell lines level of p107 was higher than in HK2, similar to colon cancer [36]. In the presence of T3 level of p107 in HK2 at G1/S is higher than in Caki-2 and Caki-1 cell lines. Lowered protein level in cancerous cell lines could be reason of faster proliferation. Triiodothyronine caused huge decrease of p107 protein level at G12/S what is probably the reason of stimulatory effect of T3 on Caki-2 and Caki-1 cells proliferation.

**Figure 7:**
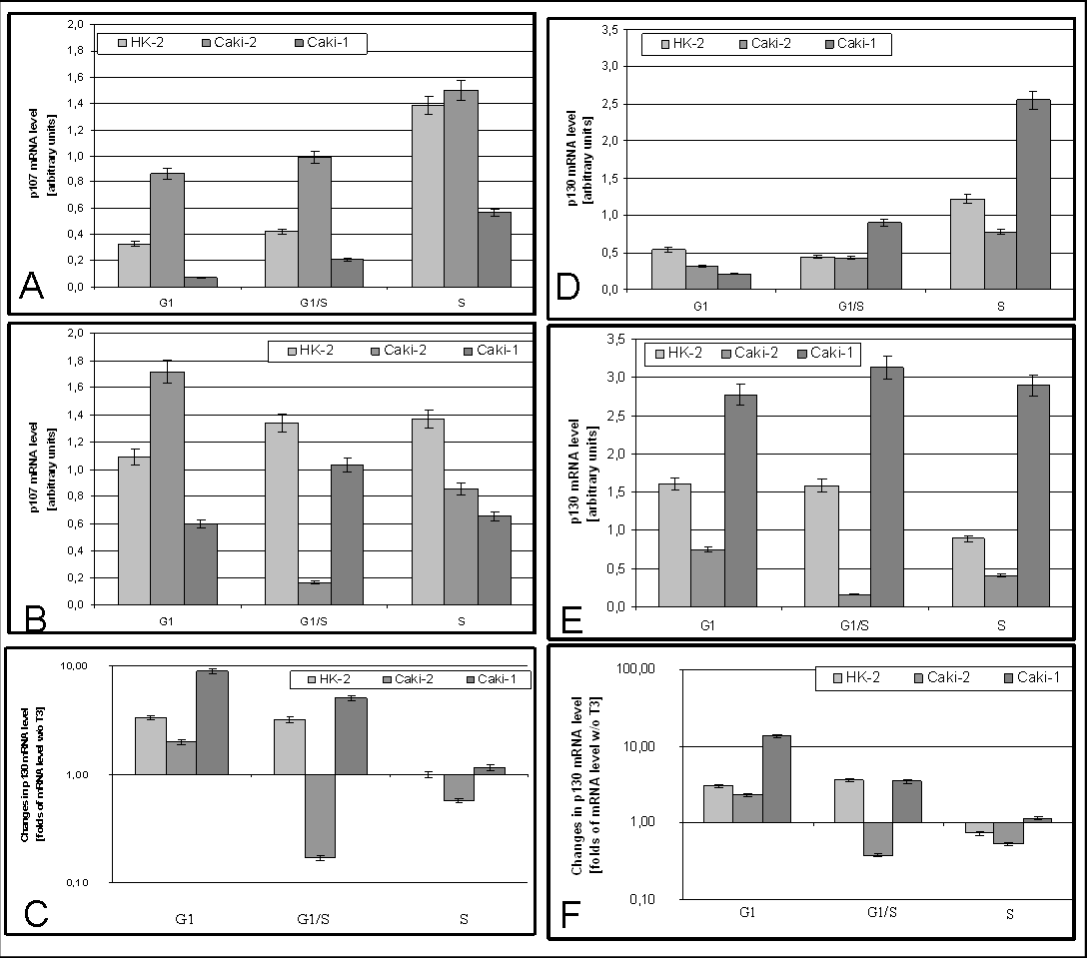
**Expression of p107 and p130 mRNA**. A) Expression of p107 in cells cultured without triiodothyronine B) Expression of p107 in cells cultured with 100 nM triiodothyronine C) Changes in p107 mRNA expression caused by 100 nM triiodothyronine D) Expression of p130 in cells cultured without triiodothyronine E) Expression of p130 in cells cultured with 100 nM triiodothyronine F) Changes in p130 mRNA expression caused by 100 nM triiodothyronine.

In HK2 cells cultured without triiodothyronine p107 mRNA was raising during cell cycle progression (G1:0,33 ± 0,016; G1/S:0,42 ± 0,021; S:1,38 ± 0,069). In Caki-2 mRNA level also increases during cell cycle progression (G1:0,86 ± 0,043 G1/S:0,99 ± 0,049 S:1,50 ± 0,075) p107 mRNA level was at G1 and G1/S higher than observed in HK2. In Caki-1 level of p107 mRNA was lower than in other lines, but also rises during cell cycle progression (G1:0,07 ± 0,003 G1/S:0,21 ± 0,010 S:0,57 ± 0,028) (Figure 7A).

In the presence of triiodothyronine level of p107 mRNA was rising during the cell cycle progression with peak at G1/S and S (G1:1,09 ± 0,055; G1/S:1,34 ± 0,067; S:1,39 ± 0,068) (Figure 7B). In Caki-2 enormous drop of mRNA p107 level was observed at G1/S (G1:1,72 ± 0,085; G1/S:0,17 ± 0,08) and rise at S (0,86 ± 0,044). In Caki-1 level of p107 mRNA was rising at G1/S and declining at S (G1:0,60 ± 0,030; G1/S:1,03 ± 0,52; S:0,66 ± 0,033). Both cancerous cell lines has lower level of p107 than HK2 (except G1 in Caki-2).

In HK2 triiodothyronine stimulates expression of p107 mRNA at G1 and G1/S, has no effect at S. In Caki-2 triiodothyronine causes rise of p107 mRNA level at G1, but at G1/S an S T3 has negative effect on this mRNA expression. In Caki-1 effects of T3 were similar to those observed in HK2 (Figure 7C).

Higher level of p107 mRNA observed in the presence of T3 in HK2 cells is probably reason of lower proliferation due to repressing effects of CERC and inhibition of cyclinE/cdk2 complexes. Reduced p107 mRNA level observed in Caki-2 is reason of faster proliferation. Triiodothyronine causes enormous drop of p107 mRNA level in Caki-2 cells which could stand for the proproliferative effects of T3.

Level of p130 mRNA in HK2 cells cultured without T3 was diminished at G1/S and doubled at S (G1:0,53 ± 0,026; G1/S:0,44 ± 0,021; S:1,22 ± 0,059) (Figure 7D). In Caki-2 level of p130 mRNA at G1 was two fold higher than In HK2, and rises during cell cycle progression (G1:0,32 ± 0,016; G1/S: 0,43 ± 0,021; S:0,77 ± 0,039). In Caki-1 mRNA p130 level at G1 was lower than in other cell lines and was rising in cell cycle progression (G1:0,21 ± 0,010; G1/S:0,90 ± 0,044; S:2,55 ± 0,120).

In the presence of T3 in HK2 cells level of p130 mRNA was unchanged at G1 and G1/S (G1:1,61 ± 0,09; G1/S: 1,59 ± 0,07 p > 0,05) and decreases at S (S:0,89 ± 0, 041) (Figure 7E). In Caki-2 p130 mRNA level was lower than in HK2, In G1/S level of this mRNA drops five fold at G1/S and rises at S (G1:0,75 ± 0,03; G1/S: 0,16 ± 0,007; S:0,41 ± 0,003). In Caki-1 p130 mRNA level was higher than In HK2 and Caki-2 cell lines, and rises at G1/S and decreases at S (G1:2,77 ± 0,149; G1/S: 3,33 ± 0,166; S:2,90 ± 0,150).

In HK2 triiodothyronine stimulates expression of p130 mRNA at G1 and G1/S and causes repression at S. In Caki-2 cells at G1 triiodothyronine stimulates expression, but in G1/S causes drop of mRNA level, effect At S was strongest than in HK2. In Caki-1 stimulates expression of p130 at all investigated parts of cell cycle(Figure 7F).

Higher than in Caki-2 level of p130 observed in HK2 could be one of the reasons of slower proliferation of HK2 cells. Lowered level of p130 was also observed in ovarian and endometrial cancer [37,38]. In the presence of T3 level of p130 was also higher in HK2 than in Caki-2, and like with p107 huge decrease of mRNA at G1./S in Caki-2 line was observed. Those two facts are probably the reason of slower HK2 and faster Caki-2 proliferation. The most important effect of triiodothyronine seems to be downregulation of p130 mRNA observed at G1/S in Caki-2 cells. That is probably the reason of different effects of T3 observed in HK2 and Caki-2.

## Conclusion

HK2 cell line proliferates slower than Caki-2 and Caki-1 lines. Those differences in cells cultured without triiodothyronine are result of lower E2F4 mRNA level in Caki-2 and Caki-1 line, and lower p130 level in Caki-2 line. In the presence of T3 faster proliferation of ccRCC cells is result of lower E2F4 level which drops during cell progression, drop of p107 at G1/S, lower p130 and decrease of its mRNA level with cell cycle progression.

Triiodothyronine has opposite effects on proliferation of HK2 and Caki-2, Caki-1 cells. It inhibits proliferation of proximal tubules cells and stimulates cancer cells proliferation. Those phenomena could be explained by Caki-2 negative regulation of E2F5, p107 and p130 expression and decrease in p107 protein level, or limited to Caki-1 negative regulation of p130 expression.

The differences could be to some extent attributed to the expression pattern of TR in different cell lines, however this hypothesis needs more detailed investigation.

In our opinion the results could help to understand role of T3 in ccRCC, and partially answer the question of changes in regulation of proliferation of cancer cells caused by disturbances in T3 signaling pathway.

## Abbreviations

CERC: cyclin E repressor motif; CERM: cyclin E repressor complex; ccRCC: clear cell renal cell carcinoma; TR: thyroid hormone receptor; TH: thyroid hormone; T3: triiodothyronine.

## Competing interests

The authors declare that they have no competing interests.

## Authors' contributions

PP performance of experiments, participated in its design and helped to draft the manuscript. AN conceived of the study, and participated in its design and coordination and helped to draft the manuscript. All authors read and approved the final manuscript.
